# Effect of statins on post-contrast acute kidney injury: a multicenter retrospective observational study

**DOI:** 10.1186/s12944-021-01489-7

**Published:** 2021-07-05

**Authors:** Maoning Lin, Tian Xu, Wenjuan Zhang, Duannbin Li, Ya Li, Xulin Hong, Yi Luan, Wenbin Zhang, Min Wang

**Affiliations:** 1 Department of Cardiovascular Diseases, Sir Run Run Shaw Hospital, College of Medicine, Zhejiang University, No 3 East of Qinchun Road, Hangzhou, Zhejiang 310000 People’s Republic of China; 2 Key Laboratory of Cardiovascular Intervention and Regenerative Medicine of Zhejiang Province, Hangzhou, 310000 People’s Republic of China; 3 Department of Information Technology, Sir Run Run Shaw Hospital, College of Medicine, Zhejiang University, No 3 East of Qingchun Road, Hangzhou, Zhejiang 310000 People’s Republic of China

**Keywords:** Post-contrast acute kidney injury, Statins, Lipid-lowering effect, Anti-inflammatory effect, Percutaneous coronary intervention, Path analysis, Mediation analysis

## Abstract

**Background:**

Post-contrast acute kidney injury (PC-AKI) is a severe complication of coronary angiography (CAG) and percutaneous coronary intervention (PCI). Currently, the effect of statins on PC-AKI and its mechanism remains unclear.

**Methods:**

This multicenter retrospective observational study included 4386 patients who underwent CAG or PCI from December 2006 to December 2019 in Sir Run Run Shaw Hospital and its medical consortium hospitals. Serum creatinine pre- or post-procedure within 72 h after PCI was recorded. Multivariate logical regression was used to explore whether preoperative use of statins was protective from PC-AKI. The path analysis model was then utilized to look for the mediation factors of statins.

**Results:**

Four thousand three hundred eighty-six patients were enrolled totally. The median age of the study population was 68 years old, 17.9% with PC-AKI, and 83.3% on preoperative statins therapy. The incidence of PC-AKI was significantly lower in group of patients on statins therapy. Multivariate regression indicated that preoperative statins therapy was significantly associated with lower percentage of elevated creatinine (β: -0.118, *P* < 0.001) and less PC-AKI (OR: 0.575, *P* < 0.001). In the preoperative statins therapy group, no statistically significant difference was detected between the atorvastatin and rosuvastatin groups (OR: 1.052, *P* = 0.558). Pathway model analysis indicated a direct protective effect of preoperative statins therapy on PC-AKI (*P* < 0.001), but not through its lipid-lowering effect (*P* = 0.277) nor anti-inflammatory effect (*P* = 0.596). Furthermore, it was found that “low-density lipoprotein cholesterol (LDL-C)→C-reactive protein (CRP)” mediated the relationship between preoperative statins therapy and PC-AKI (*P* = 0.007). However, this only explained less than 1% of the preoperative protective effects of statins on PC-AKI.

**Conclusion:**

Preoperative statins therapy is an independent protective factor of PC-AKI, regardless of its type. This protective effect is not achieved by lipid-lowering effect or anti-inflammatory effect. These findings underscore the potential use of statins in preventing PC-AKI among those at risk.

## Introduction

Post-contrast acute kidney injury (PC-AKI), defined as “an increase in serum creatinine ≥ 0.3mg/dl (26.5μmol/l), or ≥ 1.5 times the baseline value within 48–72h of exposure to a contrast medium (CM)” [1, 2], accounts for up to 30% of acute kidney injury in hospitalized patients [3]. Millions of cardiac catheterizations are currently performed every year [4], and hence the concerns about PC-AKI have grown recently. Prevention of PC-AKI remains dependent on volume expansion with sodium bicarbonate or normal saline. However, the benefit of drugs such as statins, N-acetylcysteine, angiotensin-converting enzyme inhibitors (ACEI) and ezetimibe in PC-AKI’s prevention remains unclear. According to the latest guidelines on European Society of Urogenital Radiology (ESUR) [2], there are no specific effective preventive measures of PC-AKI other than volume expansion.

Statins play an essential role in the cardiovascular field, but their effects on PC-AKI are inconsistent. There is no consensus among experts on the usefulness of pretreatment with statins. This is despite the 2014 European Society of Cardiology’s guidelines which stated that short-term and high-intensity statins therapy reduces the risk of PC-AKI in patients undergoing myocardial revascularization [5]. While some studies have reported a reduction in PC-AKI events among statins users [6–10], others did not show such benefit [11–15], and one study even demonstrated more PC-AKI outcomes with statins [13]. Given these contradicting results, current PC-AKI guidelines do not recommend statins. Nevertheless, these studies were focused on exploring the effects of short-term, high-dose statins or high plasma exposure of statins on PC-AKI.

On the other hand, up to the current knowledge, few studies addressed the association between long-term use of statins (> 8 weeks) before CM and PC-AKI. Furthermore, the most recent ESUR guidelines, mentioned that the effects of long-term statins on PC-AKI are unclear in patients undergoing CAG/PCI [2]. Therefore, one of the study’s objectives was to explore the long-term statins use’s impact before receiving CM on PC-AKI.

The pathophysiology of PC-AKI is not exactly understood, and several studies trying to fill gaps in knowledge are underway [16–19]. Three basic mechanisms have been proposed that act together on PC-AKI: reactive oxygen species formation, tubular cell toxicity, and medullary hypoxia with renal vasoconstriction [16, 17, 20, 21]. Statins, known for their lipid-lowering effects, also have other non-lipid modifiable effects, named pleiotropic effects [22], of which the most important effects are reducing oxidative stress [23–26] and anti-inflammatory [27]. The pleiotropic effect of statins may have important clinical significance in preventing PC-AKI [7]. Few prior study explored the intermediate mechanism of statins. Thus, in addition to exploring the effectiveness of long-term use of statins on PC-AKI, this study used path analysis methods based on clinical test indicators, LDL-C and CRP, to explore the specific mechanisms by which statins may favorably impact PC-AKI.

## Methods

### Study design and setting

This was a multicenter retrospective observational study. The study included 4386 consecutive patients who underwent CAG or PCI from December 2006 to December 2019 in Sir Run Run Shaw Hospital and its medical consortium hospitals. Patients with terminal-stage renal disease or under hemodialysis before the operation, patients younger than 18 years, and patients with missing serum creatinine pre- or post-procedure in 72 h after PCI were excluded. The study was approved by the Ethics Committee.

### Definitions

PC-AKI was defined according to the ESUR as an increase in serum creatinine ≥26.5 μmol/l (0.3 mg/dl) or ≥ 1.5 times the baseline value within 48–72 h of contrast medium (CM) exposure. Preoperative statins use was defined as patients with long-term statins usage more than 8 weeks before CAG or PCI. CM in excess was defined as the ratio of CM volume (ml)/eGFR (ml/min/1.73m^2^) exceeded three times, according to ESUR [1].

### Data collection

The data collected from Hospital Information System (HIS) included age, gender, body mass index (BMI), comorbidities, and current medication. The details of the procedure, and results of laboratory blood biochemical tests were documented. Serum creatinine concentrations were assessed in all patients at hospital admission. The postoperative serum creatinine concentrations recorded were the highest level measured at least 3 times within a 72- timeframe. Patients who used long-term statins before the operation were divided into two groups: rosuvastatin and atorvastatin. The endpoint was developing PC-AKI.

### Data analysis

Statistical Package for Social Sciences (SPSS version 20) was applied for all statistical analysis. *P* values < 0.05 was considered as statistically significant in this study. Continuous variables were presented as median/interquartile range and groups were compared with nonparametric tests. Categorical variables were presented as frequencies/percentages, and Chi-square analysis was used in groups. Linear regression analysis was utilized to explore the significant predictors of the percentage of elevated creatinine (continuous variable), while logistic regression analysis was utilized for exploring the significant predictors of PC-AKI (binary variable). SPSS Amos was used to analyze the direct and indirect effects of statins on PC-AKI.

## Results

### Baseline characteristics

Table 1 summarized baseline clinical characteristics stratified by PC-AKI status. A total of 4386 patients were enrolled. The median age was 68 years old, 66% were male, 63.6% had hypertension and 24.1% had diabetes. The baseline LDL cholesterol was 2.08/1.13 mmol/L, the baseline CRP was 2.3/7.13 mg/L, and 4.6% of the patients used CM in excess. About 83.3% of patients received preoperative statins therapy on admission.

**Table 1:** Baseline

	Total (*n* = 4386)	non-PC-AKI (*n* = 3599)	PC-AKI (*n* = 787)	*P*
Male, n/%	2895/66	2422/67.3	473/60.1	<0.001
Age, years	68/13	68/13	70/13	<0.001
Smoking, n/%	744/17	628/17.4	116/14.7	0.067
Drinking, n/%	674/15.4	576/16	98/12.5	0.012
Hypertension, n/%	2788/63.6	2274/63.2	514/65.3	0.261
Diabetes, n/%	1058/24.1	836/23.2	222/28.2	0.003
eGFR, mL/min/1.73 m2	84.38/28.9	84.61/27.41	83.02/38.1	0.012
Uric acid, μmol/L	364/135	364/131	364/150	0.504
LDL-C, mmol/L	2.08/1.13	2.08/1.12	2.08/1.18	0.485
CRP, mg/L	2.3/7.13	2/5.8	4.4/14.8	<0.001
LVEF, %	62/16	62.8/14.53	59.5/17.9	<0.001
Pre-operative statin therapy, n/%	3654/83.3	3051/84.8	603/76.6	<0.001
Atorvastatin, n/%	2307/63.1	1906/62.5	401/66.5	0.173
ACEI, n/%	704/16.1	565/15.7	139/17.7	0.174
Diuretic, n/%	1383/31.5	1124/31.2	259/32.9	0.359
Aspirin, n/%	3632/82.8	2969/82.5	663/84.2	0.206
Ezetimibe, n/%	275/6.3	237/6.6	38/4.8	0.066
Excess volumes of CM, n/%	185/4.6	137/4.2	48/6.6	0.005
Iso-osmolar CM, n/%	1380/31.6	1132/31.6	248/31.6	0.988
Contrast Volume, ml	80/80	80/80	80/90	0.249
Prior myocardial infarction, n/%	71/1.6	58/1.6	13/1.7	0.935
Prior PCI, n/%	230/5.2	191/5.3	May-39	0.689
Prior CABG, n/%	15/0.3	14/0.4	1/0.1	0.254
Prior cardiac surgery /except CABG, n/%	15/0.3	11/0.3	4/0.5	0.378
Angiography combined with PCI, n/%	1925/43.9	1571/43.7	354/45	0.496
Multi vessel PCI, n/%	1075/56.2	878/56.2	197/56.1	0.977
Total length of stents, mm	38/38	39/39	36/33.5	0.083

*Abbreviations*: *PC-AKI* post-contrast acute kidney injury, *eGFR* estimated glomerular filtration rate, *LDL-C* low-density lipoprotein cholesterol, *CRP C*-reactive protein, *LVEF* left ventricular ejection fractions, *CM* contrast medium, *ACEI* angiotensin converting enzyme inhibitors, *PCI* percutaneous coronary intervention, *CABG* coronary artery bypass grafting

Compared with patients in the non-PC-AKI group, patients with PC-AKI were more likely to be older (70/13 vs. 68/13; *P* < 0.001), less male (60.1% vs. 67.3%; *P* < 0.001) and drinkers (12.5% vs. 16%; *P* = 0.038). Patients in the PC-AKI group also had lower prevalence of preoperative therapy (76.6% vs. 84.8%; *P* < 0.001), lower eGFR (83.02/38.1 vs. 84.61/27.41; *P* = 0.012), lower left ventricular ejection fractions (LVEF) (59.5/17.9 vs. 62.8/14.53; *P* < 0.001), higher CRP (4.4/14.8 vs. 2/5.8, *P* < 0.001), and higher proportion of excess volumes of CM (6.6% vs. 4.2%, *P* = 0.005), and more prevalence of diabetes mellitus (28.2% vs. 23.2%, *P* = 0.003). There were no significant differences between the groups in smoking status, hypertension, uric acid, LDL-C, angiotensin converting enzyme inhibitors (ACEI), diuretic, ezetimibe, aspirin, type and volume of CM, history of myocardial infarction and cardiac surgery, type of operation, multi vessel PCI and total length of stents.

### Regression analysis

Table 2 showed the associations of different risk factors with PC-AKI using logistics regression analysis. Seven variables were significantly associated with PC-AKI, including age, male, diabetes, CRP, LVEF, excess volumes of CM and preoperative statins therapy.

**Table 2:** Univariate and multivariate logistic association for PC-AKI among the whole population

	Univariable Analysis				Multivariable Analysis			
	B	OR	95%CI	*P*	B	OR	95%CI	*P*
Male	−0.312	0.732	0.625–0.858	<0.001	− 0.397	0.672	0.558–0.809	<0.001
Age	0.025	1.025	1.018–1.033	<0.001	0.025	1.026	1.016–1.035	<0.001
Smoking	−0.201	0.818	0.66–1.014	0.067	0.003	1.003	0.771–1.304	0.984
Drinking	−0.292	0.746	0.593–0.939	0.013	−0.037	0.964	0.736–1.262	0.787
Hypertension	0.093	1.097	0.933–1.29	0.261				
Diabetes	0.261	1.299	1.092–1.545	0.003	0.203	1.226	1.012–1.484	0.037
eGFR	−0.007	0.993	0.99–0.996	<0.001	0.003	1.003	0.998–1.007	0.209
Uric acid	0	1	0.999–1.001	0.928				
LDL-C	−0.025	0.976	0.895–1.063	0.573				
CRP	0.011	1.011	1.008–1.014	<0.001	0.009	1.009	1.006–1.012	<0.001
LVEF	−0.021	0.979	0.973–0.985	<0.001	−0.023	0.977	0.971–0.984	<0.001
Excess volumes of CM	0.479	1.614	1.15–2.266	0.006	0.55	1.733	1.156–2.596	0.008
Iso-osmolar CM	−0.001	0.999	0.846–1.179	0.988				
Contrast Volume	0	1	0.999–1.001	0.557				
Pre-operative statin therapy	− 0.53	0.589	0.488–0.711	<0.001	−0.554	0.575	0.466–0.709	<0.001
Prior myocardial infarction	0.163	1.177	0.925–1.497	0.185				
Prior PCI	0.134	1.144	0.956–1.369	0.143				
Prior CABG	−0.092	0.912	0.579–1.437	0.691				
Prior cardiac surgery (except CABG)	−0.01	0.991	0.644–1.524	0.965				
Angiography combined with PCI	0.054	1.055	0.904–1.232	0.496				
Multi vessel PCI	−0.003	0.997	0.789–1.258	0.977				
Total length of stents	−0.006	0.994	0.988–1	0.071				

*Abbreviations*: *PC-AKI* post-contrast acute kidney injury, *eGFR* estimated glomerular filtration rate, *LDL-C* low-density lipoprotein cholesterol, *CRP C*-reactive protein, *LVEF* left ventricular ejection fractions, *CM* contrast medium, *ACEI* angiotensin converting enzyme inhibitors, *PCI* percutaneous coronary intervention, *CABG* coronary artery bypass grafting

Male (OR: 0.672, 95%CI: 0.558 ~ 0.809; *P* < 0.001), younger (OR: 1.026, 95%CI: 1.016 ~ 1.035; *P* < 0.001), higher LVEF (OR = 0.977, 95%CI: 0.971 ~ 0.984; *P* < 0.001) and preoperative statins (OR: 0.575, 95%CI: 0.466 ~ 0.709; *P* < 0.001) were associated with lower odds (i.e. protective) of PC-AKI, while CRP (OR: 1.009, 95%CI: 1.006 ~ 1.012; *P* < 0.001), diabetes (OR: 1.226, 95%CI: 1.012 ~ 1.484; *P* = 0.037) and excess volumes of CM (OR: 1.733, 95%CI: 1.156 ~ 2.596; *P* = 0.008) indicated higher odds (i.e. increased risk) of PC-AKI.

Furthermore, in multivariate linear regression analysis, preoperative statins therapy was associated with lower percentage of elevated creatinine levels (β: -0.118; *P* < 0.001), as shown in Table 3.

**Table 3:** Univariate and multivariate linear association for Percentage of elevated creatinine among the whole population

	Univariable Analysis			Multivariable Analysis		
	B	β	*P*	B	β	*P*
Male	−1.973	−0.024	0.109			
Age	0.234	0.065	<0.001	0.238	0.068	<0.001
Smoking	−2.232	−0.022	0.151			
Drinking	−3.535	−0.033	0.029	−1.56	−0.015	0.353
Hypertension	−0.909	−0.011	0.454			
Diabetes	3.007	0.033	0.028	2.824	0.032	0.045
eGFR	−0.027	−0.016	0.286			
Uric acid	0.003	0.009	0.562			
LDL-C	−0.335	− 0.008	0.603			
CRP	0.224	0.13	<0.001	0.176	0.106	<0.001
LVEF	−0.345	− 0.117	<0.001	−0.294	− 0.102	<0.001
Excess volumes of CM	6.616	0.036	0.022	4.567	0.025	0.11
Iso-osmolar CM	−0.027	0	0.983			
Contrast Volume	−0.002	− 0.003	0.834			
Pre-operative statin therapy	−11.39	−0.11	<0.001	−12.06	−0.118	<0.001
Prior myocardial infarction	−0.733	−0.002	0.874			
Prior PCI	−1.948	−0.011	0.457			
Prior CABG	−8.818	−0.013	0.378			
Prior cardiac surgery (except CABG)	2.18	0.003	0.827			
Angiography combined with PCI	−0.978	− 0.013	0.406			
Multi vessel PCI	−1.287	−0.019	0.416			
Total length of stents	0.001	0.001	0.972			

*Abbreviations*: *PC-AKI* post-contrast acute kidney injury, *eGFR* estimated glomerular filtration rate, *LDL-C* low-density lipoprotein cholesterol, *CRP C*-reactive protein, *LVEF* left ventricular ejection fractions, *CM* contrast medium, *ACEI* angiotensin converting enzyme inhibitors, *PCI* percutaneous coronary intervention, *CABG* coronary artery bypass grafting

In addition, as shown in Table 4, the type of statins (rosuvastatin or atorvastatin) had no significant correlation with PC-AKI.

**Table 4:** Univariate and multivariate logistic association for PC-AKI among patients with pre-operative statin therapy

	Univariable Analysis				Multivariable Analysis			
	B	OR	95%CI	*P*	B	OR	95%CI	*P*
Male	−0.3	0.741	0.618–0.888	0.001	−0.403	0.668	0.541–0.826	<0.001
Age	0.027	1.028	1.019–1.037	<0.001	0.025	1.025	1.014–1.036	<0.001
Smoking	−0.224	0.799	0.629–1.016	0.067	−0.035	0.965	0.72–1.293	0.812
Drinking	−0.316	0.729	0.563–0.944	0.017	−0.009	0.991	0.734–1.339	0.954
Hypertension	0.207	1.229	1.016–1.487	0.033	0.064	1.066	0.861–1.318	0.559
Diabetes	0.413	1.511	1.251–1.825	<0.001	0.283	1.326	1.078–1.632	0.008
eGFR	−0.008	0.992	0.989–0.996	<0.001	0.004	1.004	0.999–1.009	0.097
Uric acid	0	1	0.999–1.001	0.901				
LDL-C	0.003	1.003	0.913–1.102	0.945				
CRP	0.012	1.013	1.009–1.016	<0.001	0.01	1.011	1.007–1.014	<0.001
LVEF	−0.025	0.975	0.969–0.981	<0.001	−0.026	0.974	0.967–0.981	<0.001
Excess volumes of CM	0.571	1.77	1.241–2.526	0.002	0.615	1.85	1.205–2.84	0.005
Iso-osmolar CM	0.001	1.001	0.831–1.204	0.996				
Contrast Volume	0.001	1.001	1–1.002	0.119				
Types of statins, Atorvastatin	0.083	1.087	0.929–1.271	0.297	0.051	1.052	0.887–1.249	0.558
Prior myocardial infarction history	0.164	1.178	0.64–2.168	0.598				
Prior PCI history	0.075	1.077	0.754–1.54	0.682				
CABG history	−0.946	0.388	0.051–2.973	0.362				
Cardiac surgery history (except CABG)	0.814	2.257	0.693–7.353	0.177				
Angiography combined with PCI	0.202	1.224	1.027–1.459	0.024				
Multi vessel PCI	0.035	1.035	0.813–1.318	0.779				
Total length of stents	−0.005	0.995	0.989–1.001	0.127				

*Abbreviations*: *PC-AKI* post-contrast acute kidney injury, *eGFR* estimated glomerular filtration rate, *LDL-C* low-density lipoprotein cholesterol, *CRP C*-reactive protein, *LVEF* left ventricular ejection fractions, *CM* contrast medium, *ACEI* angiotensin converting enzyme inhibitors, *PCI* percutaneous coronary intervention, *CABG* coronary artery bypass grafting

### Path analysis

**Fig. 1:**
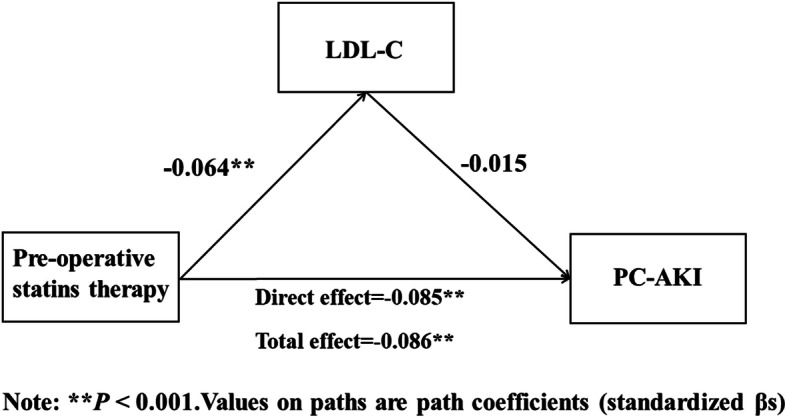
Model of the mediating effect of LDL-C on the association between Pre-operative statins therapy and PC-AKI

**Fig. 2:**
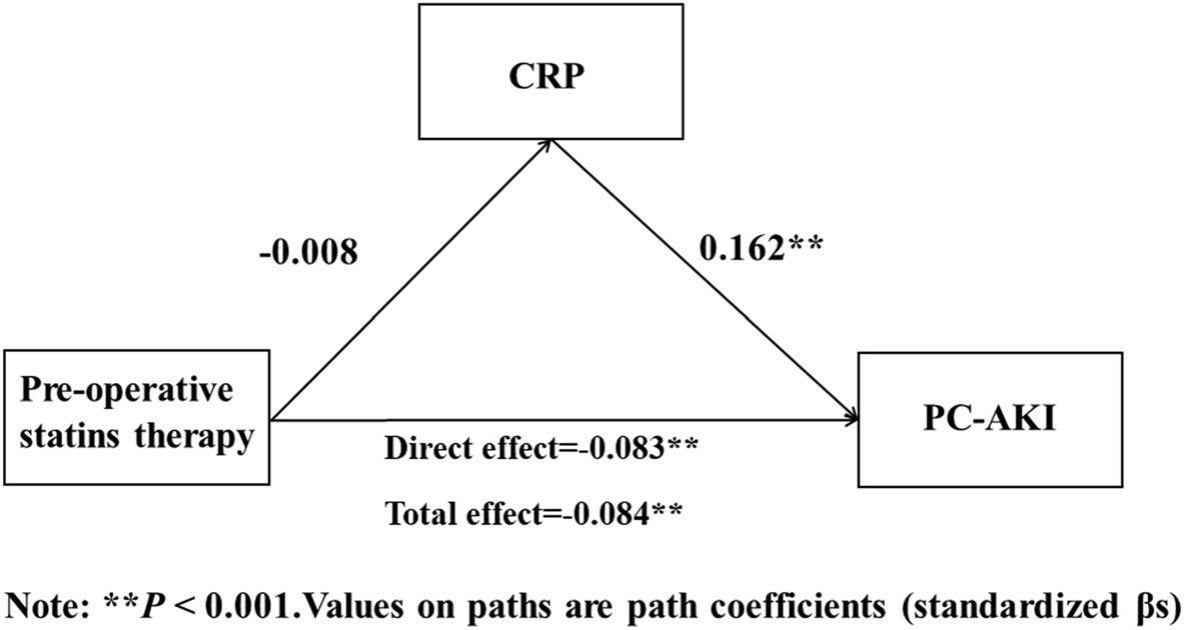
Model of the mediating effect of CRP on the association between Pre-operative statin therapy and CIAKI

**Fig. 3:**
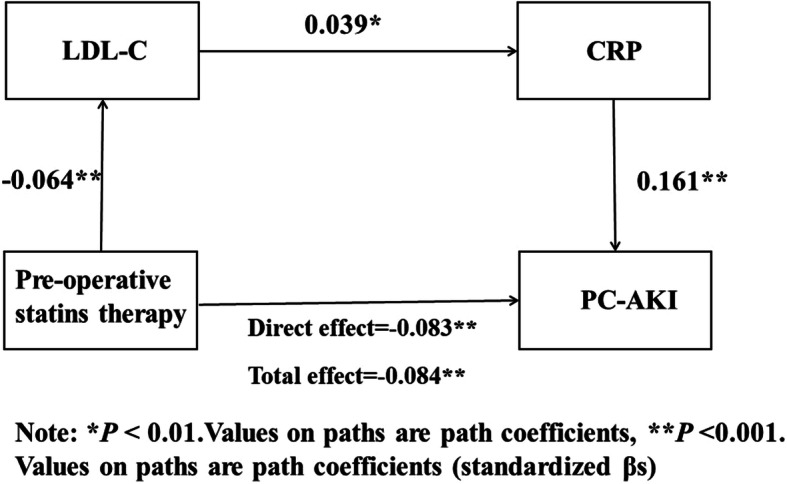
Model of the mediating effect of LDL-C, CRP on the association between Pre-operative statins therapy and PC-AKI

Structural equation modeling with observed variables in SPSS Amos was applied to test the relationships between preoperative statins therapy and PC-AKI (Figs. 1, 2, and 3), while controlling for sociodemographic variables. The results showed that preoperative statins therapy was significantly negatively associated with PC-AKI (β = −.0.085, *P* < 0.001) and LDL-C had no significant effect on PC-AKI (*P* = 0.311). This indicated that LDL-C was not a mediator for the relationship between preoperative statins therapy and PC-AKI (*P* = 0.277) (Fig. 1). In addition, CRP positively predicted PC-AKI (β = 0.162, *P* < 0.001) but CRP in patients with and without preoperative statins therapy was not significantly different (*P* = 0.601). This also indicated that CRP was not a mediator for the relationship between preoperative statins therapy and PC-AKI (*P* = 0.596) (Fig. 2). However, CRP positively predicted PC-AKI (β = 0.039, *P* = 0.009), and therefore, “LDL-C → CRP” was a partial mediator for the relationship between preoperative statins therapy and PC-AKI. The value of this effect was less than 0 and tended to 0, with a bootstrap (50,000 samples) 95% confidence intervals of − 0.001 to 0 (*P* = 0.007). Since this confidence interval did not include zero, it was concluded that there was a significant mediation effect of “LDL-C → CRP” on the relationship between preoperative statins therapy and PC-AKI. Still, it only explained < 1% effects (Fig. 3).

## Discussion

Statins are widely used as a preventive therapy for coronary artery disease, suggesting their potential usefulness in PC-AKI. The current study provided evidence that long-term use of statins independently decreased the risk of PC-AKI. This favorable effect seems to be a class effect of statins regardless of its type, and was not achieved through the statins’ lipid-lowering and anti-inflammatory mechanisms but other unknown mechanisms that need to be explored. These findings have important implications for patients undergoing CAG or PCI.

With the enhanced role of CM in the cardiovascular field, the focus on PC-AKI is expected to rise. Prevention of PC-AKI could be reflected as lower mortality, morbidity, treatment costs, and length of hospital [28, 29]. Hence, looking for preventive therapies is needed. In this regard, the role of statins in the preventing PC-AKI remains controversial due to contradicting reports [6–15]. Notably, however, recent meta-analysis [30], including 8 RCTs and 4635 patients, showed that statins pretreatment was effectively prevented PC-AKI, which is consistent with this study. The inconsistency observed among studies may be because statins lack renal protection in patients with advanced kidney disease. Differences in the proportions of patients with severe kidney disease could result in differences in conclusions. In this study, the proportion of patients with poor renal function was small. Also, few studies addressed the relevance of long-term use of statins before receiving CM and PC-AKI. Differences in the duration of receiving statins could also explain some of these inconsistencies. This study proved that those patients who received long-term use of statins would have a lower risk of PC-AKI.

The pleiotropic effect of statins may have important clinical significance in preventing PC-AKI. McCullough’s review claimed that statins exert renoprotective effects in PC-AKI via blocking the absorption of CM into renal tubular cells, reducing oxidative stress, anti-proliferation of mesangial cells and anti-inflammation [31]. In 2019, Mehran’s review suggested that that statins reduce the risk of PC-AKI via their anti-inflammatory and antioxidant properties [20]. Lipid-lowering agents like rosuvastatin, atorvastatin, and simvastatin are currently used as potential pharmacological interventions in PC-AKI animal models [18, 32, 33]. Taken all together, these reports indicate that statins could decrease PC-AKI through a variety of physiological pathways, such as modulation to nitric oxide, oxidative stress, inflammatory responses, and apoptotic processes. However, there has been few clinical research to explore the intermediate mechanism of statins.

In the current study, it was found that the lower CRP reduced PC-AKI occurrence, while statins were not associated with the CRP. Meanwhile, there was no significant correlation between LDL-C and the occurrence of PC-AKI. Thus, the protective role of statins in PC-AKI has not been achieved through lipid-lowering and anti-inflammatory effects, but other unknown mechanisms. These mechanisms could be anti-proliferative [22, 34], antioxidant [23–26], immunomodulatory [35, 36], neuroprotective [37, 38], anti-diabetes [39], which need to be explored.

### Study strengths and limitations

The current study has several strengths. This includes being a multicenter study with considerable sample size. Also, it addressed a novel and clinically relevant topic by exploring the relationship between long-term use of statins before receiving CM and the odds of PC-AKI, and the intermediate mechanisms explaining the effects of statins. Findings from this analysis might support long-term use of statins before CAG or PCI, which may contribute to lower risk of PC-AKI and subsequently lower mortality, morbidity, treatment costs and length of hospital stay.

This study has potential limitations, as well. First, it was a retrospective observational study. A double-blinded randomized-controlled trial is needed to warrant long-term statins used to prevent PC-AKI in patients undergoing CM. Second, the majority of patients received statins. Likely, statins were more frequently prescribed in patients with elevated LDL-C levels, which could generate bias. Third, vascular access has been demonstrated to affect the risk of AKI after PCI, with the radial access proven to be protective compared with the femoral access [40]. However, relevant information was not available and hence residual confounding due to this factor and others remains a possibility. Furthermore, many clinical test indicators cannot temporarily be obtained, such as anti-oxidative stress and anti-proliferation, to further explore the intermediate mechanism of statins.

## Conclusion

Preoperative statins therapy is an independent protective factor of PC-AKI, which is not affected by the type of statins and not achieved by the lipid-lowering effect or anti-inflammatory effects. Patients not on preoperative statins therapy before CAG/PCI tend to have a higher incidence of PC-AKI, which informs clinical workers to be more cautious in using the dose of contrast media, and to take preventive measures more actively in clinical practice, such as fluid rehydration. These findings suggest the potential usefulness of preoperative statins therapy in preventing PC-AKI before CAG/PCI.

## Data Availability

Definitely, the corresponding author would like to provide data for proper requests.

## Abbreviations

ESUR: European Society of Urogenital Radiology
ACEI: Angiotensin converting enzyme-inhibitors
BMI: Body mass index
CKD: Chronic kidney disease
CRP: C-reactive protein
CABG: Coronary artery bypass grafting
PCI: Percutaneous coronary intervention
HIS: Hospital Information System
PC-AKI: Post-Contrast acute kidney injury
LDL-C: Low-density lipoprotein cholesterol
CAG: Coronary angiography
CM: Contrast medium
eGFR: estimated glomerular filtration rate
SPSS: Statistical Package for Social Sciences
LVEF: Left Ventricular Ejection Fractions
